# Supplementary material for: GABAA Receptor/STEP61 Signaling Pathway May Be Involved in Emulsified Isoflurane Anesthesia in Rats
Source: Int J Mol Sci. 2020 Jun 7;21(11):4078. doi: 10.3390/ijms21114078 (PMC7312199; doi:10.3390/ijms21114078)
Supplement: Supplementary file 1 [file ijms-21-04078-s001.pdf]

Supplemental Table S1. Criteria used to score the reflexes of rats anesthetized by emulsified isoflurane (EISO).

Reflexes	Score	Observation
Palpebral reflex	3	Stimulating eyelids with cotton swabs, appearance of dodging, closing eyes, blinking
	2	Slow or diminished response
	1	No response to stimulation was observed
Corneal reflex, CR	3	Stimulation of cornea with hard stubble, appearance of obvious contractions, tremors, and blinking of eyelids
	2	Slow or diminished response
	1	No response to stimulation was observed
Perianal reflex	3	Squirting the anus of the rat with a needle, and obvious anal contractions
	2	Only weak response
	1	No response to stimulation was observed

Supplemental Table S2. Criteria used to score the anesthetic effects of EISO in rats.

Criteria	Score	Observation
Sedation score	0	Normal
	1	Mild sedation (recumbent, head down, strong palpebral reflex, and normal eye position)
	2	Moderate sedation (recumbent, head down, moderate palpebral reflex, and partial ventromedial eye rotation)
	3	Profound sedation (recumbent, head down, absence of palpebral reflex, and complete ventromedial eye rotation)
Analgesia score	0	Normal (productive flight response)
	1	Mild (exaggerated movements of limbs and trying to get up)
	2	Moderate (slight movements of the limbs and trying to get up)
	3	Profound (lack of response)
Muscle relaxation score	0	Normal
	1	Mild relaxation of leg tone
	2	Moderate relaxation of leg tone
	3	Profound relaxation of leg tone
Posture score	0	Normal
	1	Sternal recumbency
	2	Lateral recumbency
Auditory response score	0	Normal
	1	Mild decrease in response (eye movement with body movement)
	2	Moderate decrease in response (eye movement without body movement)
	3	Profound decrease in response (no movement)

Supplemental Table S3. The primer sequences of the target genes and the internal reference genes.

Gene	Primer Sequences (5′-3′)	GenBank Accession [b]	Product Size
GABA$_{A}$*α1*	F[a]: AAACCTTTAACAGCGTCAGCA<br>R[a]: CAGGAATCACTGCGTTGAGA	NM_183326.2	214
NMDANR1	F: TCTGCAACTTGATTTCCACCT<br>R: GCGTCTGAGGAAGCCTATTG	NM_001270602.1	221
NR2B	F: AAGACAAGGGCCGATTCAT<br>R: GGTCAGGGTAGAGCGACTTG	NM_012574.1	161
Oprm1	F: GATCCTCTCTTCTGCCATCG<br>R: CAGGCCGTAACACACAGTGA	NM_001038597.2	186
ERK 1/2	F: AACAAACATACCGAGGTGGAG<br>R: CTGGGATGAGCCGTATGAG	NM_053842.2	176
JNK	F: TGGTGATAGATGCGTCCAAA<br>R: ACGGCTGCCCTCTTATGAC	NM_053829.2	224
P38	F: GGCCCTGCCTTTACCATATC	NM_031020.2	214

	R: GAACGCCAGCAACTGAGATT		
NF-κB	F: CGCATCCAGACCAACAATAA<br>R: CCAGAGTTCCGGTTTACTCG	NM_199267.2	214
Notch	F: GAGATGCTCCCAGCCAAGT<br>R: TCCACAACATAGCACGTTCC	NM_001105721.1	157
STEP 61	F: ACCCGTGTTTGACTGTGTGA<br>R: AGCAGGTATTCATGGGCTGA	NM_019253.3	194
FYN	F: CTGAAATTGCCAAACCTCGT<br>R: GCACCTTGTCTGGCTGTGTA	NM_012755.1	193
β-actin	F:TCACCCACACTGTGCCCATCTATGA<br>R: CATCGGAACCGCTCATTGCCGATAG	NM_031144.3	300
$GABA^{Meth}$	F:GGCACTGAAACTATGGAGTTTTAG<br>R:GCACTCTTAAACACCAAAGAGC	NW_007905917.1	195
$NMDA^{Meth}$	F:GTGCAGACATGAAGCCTCCAG<br>R:CCTTCAGCCATATAAAGTACAGC	NW_007905728.1	330
$OPRM^{Meth}$	F:ATTCAGGTTTGTACTCTGAGA<br>R:TATATTCTCCTAGGAAACACT	NW_007905639.1	256

[a] F, forward of the target primer; R, reverse of the target primer. [b] The serial number of the target genes in the National Center for Biotechnology Information (NCBI) GenBank database.

Supplemental Table S4. Results of physiological indicators after the administration of emulsified isoflurane by intravenous infusion in SD rats.

Item	Con	FE	LD	HD	SE	*p* Value		
						Treated	Time	Tr × T
BT	38.13 a	38.50 a	37.60 b	37.60 b	0.16	<0.01	0.08	<0.01
RF	143.25 a	148.18 a	69.22 b	61.53 b	3.48	<0.01	0.32	0.06

Con, Control; FE, Fat Emulsion; LD, Low Dose; HD, High Dose; BT, Body Temperature; RF, Respiratory Frequency; Tr × T, Treated × Time; Means in the same row with different Roman letters (a and b) represent significant differences.
